# Antibiotic prescription and food allergy in young children

**DOI:** 10.1186/s13223-016-0148-7

**Published:** 2016-08-17

**Authors:** Bryan L. Love, Joshua R. Mann, James W. Hardin, Z. Kevin Lu, Christina Cox, David J. Amrol

**Affiliations:** 1 Department of Clinical Pharmacy and Outcomes Sciences, South Carolina College of Pharmacy, The University of South Carolina, 715 Sumter St., CLS 314D, Columbia, SC 29208 USA; 2 Department of Preventive Medicine, The University of Mississippi Medical Center, Jackson, MS USA; 3 Department of Epidemiology and Biostatistics, Institute for Families in Society, University of South Carolina, Columbia, SC USA; 4 Department of Internal Medicine; Division of Allergy, Asthma and Immunology, University of South Carolina School of Medicine, Columbia, SC USA

**Keywords:** Allergy, Food allergy, Antibiotics, Children, Health services research

## Abstract

**Background:**

To assess the relationship between any systemic antibiotic prescription within the first year of life and the presence of an ICD-9-CM diagnosis code for food allergy (FA).

**Methods:**

This was a matched case–control study conducted using South Carolina Medicaid administrative data. FA cases born between 2007 and 2009 were matched to controls without FA on birth month/year, sex, race/ethnicity. Conditional logistic regression was used to model the adjusted odds ratio (aOR) of FA diagnosis. All models were adjusted for presence of asthma, wheeze, or atopic dermatitis.

**Results:**

A total of 1504 cases and 5995 controls were identified. Receipt of an antibiotic prescription within the initial 12 months of life was associated with FA diagnosis in unadjusted and adjusted models (aOR 1.21; 95 % CI 1.06–1.39). Compared to children with no antibiotic prescriptions, a linear increase in the aOR was seen with increasing antibiotic prescriptions. Children receiving five or more (aOR 1.64; 95 % CI 1.31–2.05) antibiotic prescriptions were significantly associated with FA diagnosis. The strongest association was noted among recipients of cephalosporin and sulfonamide antibiotics in both unadjusted and adjusted models.

**Conclusions:**

Receipt of antibiotic prescription in the first year of life is associated with FA diagnosis code in young children after controlling for common covariates. Multiple antibiotic prescriptions are more strongly associated with increases in the odds of FA diagnosis.

## Background

In the United States, food allergy affects between 4 and 8 % of children and is most prevalent during the first years of life. From 1997 to 2007, the prevalence of reported food allergy increased 18 % among children under age 18 years [1]. In a prospective study of 480 consecutive newborns followed through their third birthday, nearly 8 % of the cohort was food allergic [2]. Food allergy contributes significantly to healthcare utilization with an estimated average of 317,000 ambulatory visits per year to emergency departments or physician offices [3]. A threefold increase in overall hospitalizations related to food allergy has been reported from 1998 to 2000 through 2004–2006 [1, 3].

Newborn children need exposure to immunogenic material to develop a healthy, functional immune system. Changes in the composition, richness, and abundance of microbiota that colonize the human gut during infancy has been theorized to play a role in development in atopic disease, including food allergen sensitization [4]. Commensal bacteria have been shown to protect against food allergen sensitization in mice [5]. Attempts to translate these findings to early infancy in one study revealed that infants with low gut microbiota richness and elevated proportion of *Enterobacteriaceae* relative to *Bacteroides* spp. were associated with subsequent food allergen sensitization at 1 year of age [6]. In this study, we sought to explore the association between antibiotic prescription in the first year of life and the presence of any food allergy diagnosis code in young children.

## Patients and methods

### Study design, data source, and participant selection

We performed a matched case–control study using Medicaid billing data for children from birth to age 3 in 2010 were obtained from the South Carolina Office of Research and Statistics, a clearinghouse for administrative and vital records data in South Carolina (SC). Cases were identified as those children in 2010 with an office visit or hospitalization associated with specific ICD-9-CM codes attributed to food allergy (Table 1). Controls were selected from among children from birth to age 3 who were enrolled in SC Medicaid in 2010 and never had a diagnosis of food allergy documented in Medicaid billing records since their birth. Four controls were matched to each case using birth month, birth year, sex and race/ethnicity. Both cases and controls were limited to children who were enrolled in SC Medicaid for the entirety of their first year of life.

**Table 1:** ICD-9-CM codes used to identify food allergy hospitalizations and office visits

Description	ICD-9-CM Code	Proportion of cases with ICD-9-CM code^a^ (%)
Allergic rhinitis attributable to food	477.1	4.39
Allergic gastroenteritis and colitis	558.3	8.64
General food allergy according to specific type of food		
Peanuts	V15.01	4.65
Milk products	V15.02	6.58
Eggs	V15.03	5.12
Seafood	V15.04	1.93
Other foods	V15.05	4.85
Contact dermatitis attributable to food in contact with skin	692.5	6.18
Dermatitis attributable to food taken internally	693.1	37.37
Allergic urticaria	708.0	33.24
Anaphylactic shock attributable to adverse food reaction, specifically for:		
Unspecified food	995.60	1.46
Peanuts	995.61	1.93
Crustaceans	995.62	0.4
Fruits and vegetables	995.63	0.13
Tree nuts and seeds	995.64	0.53
Fish	995.65	0.4
Food additives	995.66	0
Milk products	995.67	0.53
Eggs	995.68	0.66
Other specified food	995.69	0.13
Other adverse food reactions, not elsewhere classified	995.7	3.32

^a^Patients may have multiple food allergy diagnoses; therefore, these percentages total >100 %

### Exposure and covariate data

The primary exposure of interest was systemic antibiotic prescription in the first year of life. Information about all antibiotic and epinephrine prescriptions in the first year of life was obtained, along with the timing of the purchase since birth, from pharmacy billing records submitted to Medicaid. A dichotomous variable was created which indicated whether a child had at least one antibiotic prescription billed during the first 12 months of life. In addition, variables indicating the total number of antibiotic prescriptions filled in the first 12 months were created. Additional analyses categorized antibiotics by class using the American Hospital Formulary System classification. Models were also estimated by creating categorical variables indicating the number of antibiotic prescriptions filled (1, 2, 3, 4, and ≥5) compared to the referent group, no antibiotic prescriptions filled. Prior to estimating any regression models, antibiotic exposure for cases was limited to prescriptions that were filled prior to the first diagnosis of food allergy.

### Statistical analysis

Using conditional logistic regression to account for matching, we estimated the association of antibiotic prescription and presence of FA diagnosis by using odds ratios (ORs) with 95 % CIs. Bivariate, conditional logistic regression was initially performed to identify significant variables (p < 0.05) associated with case status. Variables tested included a dichotomous variable for receipt of any antibiotics in the first 12 months, labor and delivery factors (e.g. antibiotics or steroids administered during childbirth), maternal factors (e.g. maternal age, smoking status prior to or during pregnancy), and child factors (e.g. neonatal intensive care admission, gestational age, presence of asthma, wheeze, or atopic dermatitis). Significant variables were then used to examine the association between any antibiotic prescription and case status using multivariable, conditional logistic regression models.

We performed several sensitivity analyses. We examined assumptions about the sensitivity and specificity FA diagnosis using ICD-9-CM coding. We repeated unadjusted and adjusted conditional logistic regression models only when the FA diagnosis code was submitted by an allergy or immunology provider. Additionally, we developed a model where only cases with epinephrine prescription (and corresponding matching controls) were considered. Finally, we considered a model that excluded diagnoses that were felt to have lower sensitivity or specificity, dermatitis, rhinitis, and urticaria. All data analyses were performed using Stata/IC statistical software, version 14.1 (StataCorp, College Station, Texas). Hypothesis tests were 2-sided with a type I error of 0.05.

### Availability of data and supporting materials

Data utilized for this study was obtained via the South Carolina Revenue and Fiscal Affairs Office, and information on obtaining data is available through their website (http://rfa.sc.gov/healthcare). The South Carolina Data Oversight Council, a public/private body formed by the state statute, oversees the collection and release of health information collected under §44-6-170, South Carolina Code of Law. The Data Oversight Council reviews and approves applications for the release of health care data at the encounter level. The authors are not permitted to share the raw data through a data use agreement.

### Ethics statement

The University of South Carolina Institutional Review Board approved the study (protocol #00017038).

## Results

### Sample characteristics

The final study sample included 5995 controls and 1504 cases with at least one food allergy related diagnosis code. The relative proportion of each ICD-9-CM diagnosis code among cases is presented in Table 1. Key baseline clinical characteristics of the sample according to food allergy diagnosis are presented in Table 2. As expected, significantly more cases had concomitant asthma, atopy, and wheeze (p < 0.001 for all). In the control group tobacco abuse during and before pregnancy was significantly more common (p < 0.05). Vaginal birth was the preferred delivery approach in the control group, and breastfeeding upon hospital discharge was more common in the case group. Only 15 % of children with FA diagnosis had a prescription for epinephrine filled by a pharmacy. The diagnosis of FA was made by an allergy or immunology provider in 20 % of FA cases.

**Table 2:** Baseline clinical characteristics

Characteristic	Cases (n = 1504)	Controls (n = 5995)	p value
Male, %	55.9	55.8	0.98
Month of birth, %			
Jan	8.58	8.56	1.00
Feb	7.85	7.86	
Mar	7.91	7.91	
Apr	6.32	6.34	
May	7.58	7.56	
Jun	8.98	8.96	
Jul	10.7	10.68	
Aug	8.44	8.46	
Sep	10.04	10.06	
Oct	7.78	7.77	
Nov	6.38	6.41	
Dec	9.44	9.46	
Year of birth, %			
2007	25.9	25.9	1.00
2008	33.4	33.5	
2009	40.7	40.6	
Race/ethnicity, %			
Caucasian	42.6	42.7	1.00
AA	40.5	40.6	
Hispanic	8.8	8.8	
American Indian	0.1	0.1	
Asian	0.5	0.5	
Unknown	7.3	7.2	
Other	0.2	0.1	
Birth weight, grams^a^	3168.1	3133.5	0.06
Asthma, %	8.4	2.0	<.001
Atopic dermatitis, %	32.8	6.7	<.001
Wheeze, %	17.1	7.3	<.001
Vaginal birth, %	60.0	62.6	0.05
Breastfeeding at hospital discharge, %	56.3	49.7	<.001
WIC status, %	80.6	79.7	0.42
NICU admission, %	6.6	6.7	0.94
Tobacco pre-pregnancy, %	17.4	19.7	0.04
Tobacco during pregnancy, %	14.0	16.5	0.02
Urban residence, %	74.2	71.2	0.02
Gestational age, weeks^a^	38.3	38.2	0.07
Maternal age, years^a^	24.5	24.1	0.01
Diabetes (gestational), %	4.6	4.5	0.91
Diabetes (pre-pregnancy), %	2.1	1.0	0.001
Antibiotics during labor/delivery, %	22.8	21.0	0.13
Antibiotics received due to abnormal conditions at birth, %	3.6	3.3	0.65
Steroids during childbirth, %	0.6	0.9	0.21

^a^Numeric variables presented as mean

Dermatitis (n = 655) was the most commonly reported food allergy related diagnosis. Allergic gastroenteritis and colitis (n = 130), anaphylaxis (n = 87) and other unspecified allergy (n = 50) were less commonly diagnosed. Specific allergies to milk (n = 99), egg (n = 77), peanut (n = 70), and seafood (n = 29) were also reported, but the majority of cases did not have a specific food allergen identified.

### Antibiotic prescriptions

A total of 9324 antibiotic prescriptions were dispensed in cases and controls. The overall proportion of patients receiving antibiotics by class were as follows: penicillin (38.2 %), cephalosporin (15.1 %), macrolide (13.1 %), and sulfonamide (5.4 %) antibiotics. These antibiotic classes represented nearly all antibiotics prescribed. The mean number of antibiotic prescriptions received was 1.53 for cases and 1.17 for controls (p < 0.001). Significantly more controls did not receive any antibiotic course during the first year of life compared to cases with food allergy, 56.6 vs. 52.0 respectively (p = 0.001). Among those receiving antibiotics, the mean number of days to first antibiotic prescription was not significantly different between cases and controls, 179 and 182 days, respectively (p = 0.33).

### Association of antibiotic prescription and Food allergy diagnosis

Receipt of an antibiotic prescription within the initial 12 months of life was associated with FA diagnosis in unadjusted models (OR 1.22; 95 % CI 1.08–1.37) (Table 3). Additionally, after adjusting for vaginal birth, breastfeeding status, gestational age, maternal age, smoking status, urban residence, pre-pregnancy diabetes, and presence of asthma, wheeze, or atopic dermatitis, the association between receipt of an antibiotic prescription and FA diagnosis remained unchanged (see Table 4). We also modeled specific types of food allergy based on available diagnostic coding; however, only peanut allergy diagnosis (aOR 2.10; 95 % CI 1.04–4.22) was associated with children receiving antibiotic prescription. Compared to children with no antibiotic prescriptions, a linear increase in the aOR was seen with increasing antibiotic prescriptions. Children receiving three (aOR 1.31; 95 % CI 1.01–1.70), four (aOR 1.43; 95 % CI 1.06–1.92), or five plus (aOR 1.64; 95 % CI 1.31–2.05) antibiotic prescriptions were significantly associated with FA diagnosis. In both unadjusted and adjusted models, the strongest association was noted among recipients of cephalosporin and sulfonamide antibiotics (Table 5).

**Table 3:** Unadjusted conditional logistic regression analyses assessing the association between antibiotic exposure and food allergy diagnosis

Variable	Reference	Unadjusted analyses		
		Odds Ratio	95 % CI	p value
Primary independent variable				
Antibiotic exposure during 1st year of life	No antibiotics	1.22	1.08–1.37	0.001
Child factors				
Breastfed	Non-breastfed	1.31	1.57–1.48	<0.001
NICU admission	Routine birth	0.98	0.77–1.25	0.90
Gestational age	By weeks	1.03	1.00–1.06	0.06
WIC enrollment	Not enrolled	1.06	0.91–1.23	0.46
Asthma, wheeze, or atopic dermatitis	Absence of these conditions	5.07	4.44–5.79	<0.001
Maternal factors				
Smoking status (pre-pregnancy)	Non-smoking status	0.85	0.72–0.99	0.03
Smoking status (during pregnancy)	Non-smoking status	0.81	0.69–0.96	0.01
Maternal age	By decade	1.16	1.05–1.30	<0.01
Diabetes (gestational)	No diabetes	0.98	0.74–1.30	0.89
Diabetes (pre-pregnancy)	No diabetes	2.10	1.33–3.31	0.001
Urban residence	Rural residence	1.16	1.02–1.32	0.02
Labor and delivery factors				
Vaginal birth	Caesarian birth	0.89	0.79–1.00	0.05
Antibiotics received due to abnormal conditions at birth	No antibiotics	1.08	0.78–1.49	0.63
Antibiotics during childbirth	No antibiotics	1.10	0.95–1.27	0.19
Steroids during childbirth	No steroids	0.59	0.28–1.25	0.17

**Table 4:** Sensitivity analyses

Model^a^	Cases^b^	Controls^b^	Unadjusted model			Adjusted model^a^		
			Odds ratio	95 % CI	p value	Odds ratio	95 % CI	p value
Model 1: original model	1504	5995	1.22	1.08–1.37	<0.01	1.21	1.06–1.39	<0.01
Model 2: diagnosis by allergy or immunology provider^c^	483 (32.1)	1926 (32.1)	1.15	0.93–1.41	0.19	1.29	0.98–1.70	0.07
Model 3: reduced model^d^	818 (54.4)	3265 (54.5)	1.22	1.04–1.43	0.01	1.24	1.03–1.50	0.02
Model 4: epinephrine prescription^e^	226 (15.0)	899 (15.0)	2.89	2.08–4.02	<0.001	2.91	1.96–4.32	<0.001

^a^All models adjusted for vaginal birth, breastfeeding, gestational age, asthma, eczema, wheeze, tobacco pre- and during pregnancy, maternal age, pre-pregnancy diabetes, and urban residence

^b^Parentheses indicates the proportion of patients retained from the full data set

^c^Only patients from the original model with food allergy diagnosed by allergy or immunology provider are included

^d^Patients with diagnoses of allergic gastroenteritis and colitis, allergic urticaria, or allergic rhinitis attributable to food are excluded

^e^Model includes only cases with epinephrine prescription and corresponding matching controls. Two controls also received epinephrine prescription for unknown reasons

**Table 5:** Unadjusted and adjusted conditional logistic regression analyses assessing the association between antibiotic drug classification and food allergy diagnosis

Antibiotic Classification	Unadjusted model			Adjusted model^a^		
	Odds ratio	95 % CI	p value	Odds ratio	95 % CI	p value
Cephalosporins	1.62	1.40–1.88	<0.001	1.50	1.27–1.77	<0.001
Macrolides	1.34	1.14–1.57	<0.001	1.36	1.13–1.63	0.001
Penicillins	1.21	1.07–1.36	<0.01	1.19	1.04–1.36	0.01
Sulfonamides	1.61	1.29–2.02	<0.001	1.54	1.19–2.01	<0.01

Antibiotics categorized according to American Hospital Formulary Service (AHFS) classification

^a^Adjusted for vaginal birth, breastfeeding, gestational age, asthma, eczema, wheeze, tobacco pre- and during pregnancy, maternal age, pre-pregnancy diabetes, and urban residence

### Sensitivity analyses

Table 5 summarizes several sensitivity analyses compared to the original bivariate and multivariate models (Model 1). The odds of antibiotic exposure were not significant when limiting the analysis to FA cases with diagnosis by allergy or immunology provider (aOR 1.29; 0.98–1.70). The results were unchanged from the original model for both unadjusted and adjusted models (aOR 1.24; 95 % CI 1.03–1.50) when diagnoses of allergic gastroenteritis, allergic urticaria, and allergic rhinitis attributable to food were removed. The odds of antibiotic exposure were significantly increased relative to the original model when only cases with epinephrine prescription were considered (aOR 2.91; 95 % CI 1.96–4.32).

## Discussion

In our sample of children enrolled in the South Carolina Medicaid Program, we identified an association between antibiotic prescription and presence of food allergy diagnosis code in young children. Children with diagnosis of food allergy received significantly more antibiotics than controls even though we censored further antibiotics after the initial FA diagnosis in the case group. Our approach could have reduced the observed association between antibiotics and food allergy, since controls had an unlimited number of antibiotic exposures up to the end of month 12, while for cases the potential for exposure continued only until the initial food allergy diagnosis. However, we felt it was important to enforce this restriction since considering antibiotic prescriptions filled after the initial food allergy diagnosis as potential risk factors would have been nonsensical.

In our study, increasing quantity of antibiotic prescriptions was associated with a higher incidence of food allergy. We theorize that this could affect the likelihood of food allergen sensitization in several ways. Multiple courses of antibiotics increase the likelihood that children are on an antibiotic while introducing a new food. Secondly, there are more opportunities for microbiota dysbiosis with more frequent antibiotic administration.

Commensal gut flora serves an important role in both immune system development and immune tolerance. Studies with germ free animals show impaired humoral and cell mediated immune function [7, 8]. Also, the interaction with normal gut flora is important in the development of regulatory T-cells and IgA antibody, both of which are important in developing tolerance to foreign proteins such as food [9, 10]. Emerging evidence suggests that alterations in commensal flora due to early antimicrobial use may increase the risk of obesity, inflammatory bowel disease, and allergic diseases [11–18]. Recent articles suggest that even antimicrobial food additives or antimicrobials in health care products may be associated with increased allergic disease [19, 20]. In addition, children born via Caesarian section may have increased food allergy in addition to other atopic disease, possibly due to the lack of early colonization with beneficial bacteria from the mother’s urogenital tract [21, 22].

Conflicting findings are available for the few studies which have examined the association between antibiotic exposure and diagnosis of food allergy. In one small retrospective case control study (n = 99 cases) utilizing the same ICD-9-CM diagnoses codes as our study, neither postnatal nor perinatal antibiotic exposure was associated with subsequent food allergy diagnosis [23]. Another study was designed to look at several allergic diseases and was not powered to identify food allergy specifically. Of the 3306 children studied, 173 had food allergy at age 4, and 230 had food allergy at age 8. While antibiotic use was associated with food allergy, the association was not significant in adjusted models [24]. More recently, in a large nested case–control study (n = 16,237) maternal exposure to antibiotics and increasing antibiotic exposure in children were associated with cow’s milk allergy [25]. Our results are consistent with these previous findings, and suggest that other types of food allergen sensitization besides cow’s milk may be associated with antibiotic exposure early in life.

Infections represent one of the most frequently encountered diseases in childhood, and prescriptions for systemic antimicrobials account for about 30 % of all prescriptions in pre-school children. In the US, children aged 3 months to 3 years of age received, on average, 2.2 antimicrobial prescriptions per year between 1996 and 2000 [26], and between 10 and 20 courses of antibiotics by the age of 18 [27]. Most of these prescriptions are for otitis media, although most cases are caused by viral infection. Additionally, some studies have suggested high rates of antimicrobial prescribing for viral infections such as bronchitis and upper respiratory infection. In one study, 44 % of children with common colds, 46 % of those with upper respiratory infections, and 75 % of children with bronchitis were treated with antibiotics [28]. Data suggests that this pattern continues despite educational efforts and increasing reports of bacterial resistance [29–31]. Antibiotics are not devoid of adverse effects: Nausea, vomiting, diarrhea, and rash are common and well known short-term side effects reported in children receiving antimicrobial agents. Antibiotics are known to affect the GI flora, with the potential to cause antibiotic–induced diarrhea or *Clostridium difficile* associated disease [32–35]. Antibiotics with broader spectrum of activity are more likely to have an impact on commensal flora than narrower spectrum agents. We are just beginning to understand the potential long-term effects of microbiotia dysbiosis due to antibiotic exposure.

Strengths of our study include matching on demographic variables including month and year of birth, multivariate analysis that controlled for variables previously associated with food allergen sensitization in other studies, and a large sample of FA children; however, our study has several limitations. First, we defined food allergy using ICD-9 codes; thus, we cannot determine whether patients had food allergy versus food intolerance. The only definitive diagnostic test for food allergy is a double-blind, placebo-controlled challenge [36], but this is not frequently done in clinical practice. Additionally, there is not one single diagnostic code for food allergy, but rather several codes exist which define the allergen or clinical presentation of allergy. In clinical practice, inconsistent or varied use of diagnosis codes between providers could lead to under or over reporting of true food allergy. On the other hand, we chose FA diagnosis codes for our study based upon prior retrospective analyses of administrative records, and we conducted sensitivity analyses comparing diagnosis by provider type, receipt of epinephrine, and exclusion of diagnoses to examine this. Second, children at risk for food allergy often have other associated allergic diseases; therefore, such children may receive more antibiotics simply because of these illnesses. Atopic dermatitis predisposes to recurrent skin infections such as impetigo and *Staphylococcal* abscesses, and asthma exacerbations or wheezing episodes are often treated with antibiotics. We controlled for asthma, wheezing and atopic dermatitis in all logistic regression models to account for this. Finally, our study population consisted of patients receiving Medicaid benefits, thus our study may be more representative of a lower socioeconomic group than the general population. Lower socioeconomic status has been associated with higher rates of asthma and allergic rhinitis [37], but recent data cite higher rates of peanut sensitization among higher socioeconomic families [38–40]. Additional investigation in a more socioeconomically diverse population would also be beneficial.

## Conclusion

In summary, we identified an association between antibiotic prescription and presence of food allergy diagnosis code in young children. Our results require cautious interpretation due to the limitations as discussed; however, our findings offer a plausible hypothesis for increasing food allergy prevalence since increased antibiotic use is common in many westernized countries. Further research is needed to examine this association in larger, more diverse patient populations with confirmed food allergy.

## Abbreviations

FA: food allergy
ICD-9-CM: International classification of diseases, ninth revision, clinical modification
OR: odds ratio
aOR: adjusted odds ratio

## References

[1] Branum AM, Lukacs SL. Food allergy among U.S. children: trends in prevalence and hospitalizations. NCHS Data Brief. 2008;(10):1–8. 19389315

[2] Bock SA (1987). Prospective appraisal of complaints of adverse reactions to foods in children during the first 3 years of life. Pediatrics.

[3] Branum AM, Lukacs SL (2009). Food allergy among children in the United States. Pediatrics.

[4] Molloy J, Allen K, Collier F, Tang ML, Ward AC, Vuillermin P (2013). The potential link between gut microbiota and IgE-mediated food allergy in early life. Int J Environ Res Public Health.

[5] Stefka AT, Feehley T, Tripathi P, Qiu J, McCoy K, Mazmanian SK, Tjota MY, Seo GY, Cao S, Theriault BR (2014). Commensal bacteria protect against food allergen sensitization. Proc Natl Acad Sci USA.

[6] Azad MB, Konya T, Guttman DS, Field CJ, Sears MR, HayGlass KT, Mandhane PJ, Turvey SE, Subbarao P, Becker AB (2015). Infant gut microbiota and food sensitization: associations in the first year of life. Clin Exp Allergy.

[7] Shreiner A, Huffnagle GB, Noverr MC (2008). The “Microflora Hypothesis” of allergic disease. Adv Exp Med Biol.

[8] Kelly D, Conway S, Aminov R (2005). Commensal gut bacteria: mechanisms of immune modulation. Trends Immunol.

[9] Tsuji M, Suzuki K, Kinoshita K, Fagarasan S (2008). Dynamic interactions between bacteria and immune cells leading to intestinal IgA synthesis. Semin Immunol.

[10] Geuking MB, Cahenzli J, Lawson MA, Ng DC, Slack E, Hapfelmeier S, McCoy KD, Macpherson AJ (2011). Intestinal bacterial colonization induces mutualistic regulatory T cell responses. Immunity.

[11] Hill DJ, Hosking CS (2004). Food allergy and atopic dermatitis in infancy: an epidemiologic study. Pediatr Allergy Immunol.

[12] Ajslev TA, Andersen CS, Gamborg M, Sørensen TI, Jess T (2011). Childhood overweight after establishment of the gut microbiota: the role of delivery mode, pre-pregnancy weight and early administration of antibiotics. Int J Obes (Lond).

[13] Shaw SY, Blanchard JF, Bernstein CN (2010). Association between the use of antibiotics in the first year of life and pediatric inflammatory bowel disease. Am J Gastroenterol.

[14] Hviid A, Svanström H, Frisch M (2011). Antibiotic use and inflammatory bowel diseases in childhood. Gut.

[15] Foliaki S, Pearce N, Björkstén B, Mallol J, Montefort S, von Mutius E (2009). Group ISoAaAiCPIS: antibiotic use in infancy and symptoms of asthma, rhinoconjunctivitis, and eczema in children 6 and 7 years old: International study of asthma and allergies in childhood phase III. J Allergy Clin Immunol.

[16] Marra F, Marra CA, Richardson K, Lynd LD, Kozyrskyj A, Patrick DM, Bowie WR, Fitzgerald JM (2009). Antibiotic use in children is associated with increased risk of asthma. Pediatrics.

[17] Murk W, Risnes KR, Bracken MB (2011). Prenatal or early-life exposure to antibiotics and risk of childhood asthma: a systematic review. Pediatrics.

[18] Risnes KR, Belanger K, Murk W, Bracken MB (2011). Antibiotic exposure by 6 months and asthma and allergy at 6 years: findings in a cohort of 1,401 US children. Am J Epidemiol.

[19] Bertelsen RJ, Longnecker MP, Løvik M, Calafat AM, Carlsen KH, London SJ, Lødrup Carlsen KC (2013). Triclosan exposure and allergic sensitization in Norwegian children. Allergy.

[20] Savage JH, Matsui EC, Wood RA, Keet CA (2012). Urinary levels of triclosan and parabens are associated with aeroallergen and food sensitization. J Allergy Clin Immunol.

[21] Bager P, Wohlfahrt J, Westergaard T (2008). Caesarean delivery and risk of atopy and allergic disease: meta-analyses. Clin Exp Allergy.

[22] Xu B, Pekkanen J, Hartikainen AL, Järvelin MR (2001). Caesarean section and risk of asthma and allergy in adulthood. J Allergy Clin Immunol.

[23] Dowhower Karpa K, Paul IM, Leckie JA, Shung S, Carkaci-Salli N, Vrana KE, Mauger D, Fausnight T, Poger J (2012). A retrospective chart review to identify perinatal factors associated with food allergies. Nutr J.

[24] Mai XM, Kull I, Wickman M, Bergström A (2010). Antibiotic use in early life and development of allergic diseases: respiratory infection as the explanation. Clin Exp Allergy.

[25] Metsälä J, Lundqvist A, Virta LJ, Kaila M, Gissler M, Virtanen SM (2013). Mother’s and offspring’s use of antibiotics and infant allergy to cow’s milk. Epidemiology.

[26] Finkelstein JA, Metlay JP, Davis RL, Rifas-Shiman SL, Dowell SF, Platt R (2000). Antimicrobial use in defined populations of infants and young children. Arch Pediatr Adolesc Med.

[27] Sharland M, Subgroup SP (2007). The use of antibacterials in children: a report of the Specialist Advisory Committee on Antimicrobial Resistance (SACAR) Paediatric Subgroup. J Antimicrob Chemother.

[28] Nyquist AC, Gonzales R, Steiner JF, Sande MA (1998). Antibiotic prescribing for children with colds, upper respiratory tract infections, and bronchitis. JAMA.

[29] Coco AS, Horst MA, Gambler AS (2009). Trends in broad-spectrum antibiotic prescribing for children with acute otitis media in the United States, 1998–2004. BMC Pediatr.

[30] de Jong J, van den Berg PB, Visser ST, de Vries TW, de Jong-van den Berg LT (2009). Antibiotic usage, dosage and course length in children between 0 and 4 years. Acta Paediatr.

[31] Bourgeois FC, Linder J, Johnson SA, Co JP, Fiskio J, Ferris TG (2010). Impact of a computerized template on antibiotic prescribing for acute respiratory infections in children and adolescents. Clin Pediatr (Phila).

[32] Beaugerie L, Petit JC (2004). Microbial-gut interactions in health and disease. Antibiotic-associated diarrhoea. Best Pract Res Clin Gastroenterol.

[33] Bishara J, Peled N, Pitlik S, Samra Z (2008). Mortality of patients with antibiotic-associated diarrhoea: the impact of Clostridium difficile. J Hosp Infect.

[34] De La Cochetière MF, Durand T, Lalande V, Petit JC, Potel G, Beaugerie L (2008). Effect of antibiotic therapy on human fecal microbiota and the relation to the development of Clostridium difficile. Microb Ecol.

[35] Freeman J, Wilcox MH (1999). Antibiotics and Clostridium difficile. Microbes Infect.

[36] Boyce JA, Assa’ad A, Burks AW, Jones SM, Sampson HA, Wood RA, Plaut M, Cooper SF, Fenton MJ, Arshad SH (2010). Guidelines for the diagnosis and management of food allergy in the United States: report of the NIAID-sponsored expert panel. J Allergy Clin Immunol.

[37] Almqvist C, Pershagen G, Wickman M (2005). Low socioeconomic status as a risk factor for asthma, rhinitis and sensitization at 4 years in a birth cohort. Clin Exp Allergy.

[38] Branum AM, Simon AE, Lukacs SL (2012). Among children with food allergy, do sociodemographic factors and healthcare use differ by severity?. Matern Child Health J.

[39] Bloom B, Cohen RA, Freeman G. Summary health statistics for U.S. children: National health interview survey, 2009. Vital Health Stat 10. 2010;(247):1–82. 21563639

[40] Yip S, Yamane G, Rans T, Quinn J, Butler J. Peanut sensitization: a circumstance of affluence in children. In: American College of Allergy, Asthma & Immunology Annual Scientific Meeting: 2012; Anaheim; 2012:P278.

